# Breast Cancer Cell Line Aggregate Morphology Does Not Predict Invasive Capacity

**DOI:** 10.1371/journal.pone.0139523

**Published:** 2015-09-29

**Authors:** Michelle J. Ziperstein, Asja Guzman, Laura J. Kaufman

**Affiliations:** Department of Chemistry, Columbia University, New York, New York, United States of America; University of Oklahoma Health Sciences Center, UNITED STATES

## Abstract

To invade and metastasize to distant loci, breast cancer cells must breach the layer of basement membrane surrounding the tumor and then invade through the dense collagen I-rich extracellular environment of breast tissue. Previous studies have shown that breast cancer cell aggregate morphology in basement membrane extract correlated with cell invasive capacity in some contexts. Moreover, cell lines from the same aggregate morphological class exhibited similarities in gene expression patterns. To further assess the capacity of cell and aggregate morphology to predict invasive capacity in physiologically relevant environments, six cell lines with varied cell aggregate morphologies were assessed in a variety of assays including a 3D multicellular invasion assay that recapitulates cell-cell and cell-environment contacts as they exist *in vivo* in the context of the primary breast tumor. Migratory and invasive capacities as measured through a 2D gap assay and a 3D spheroid invasion assay reveal that breast cancer cell aggregate morphology alone is insufficient to predict migratory speed in 2D or invasive capacity in 3D. Correlations between the 3D spheroid invasion assay and gene expression profiles suggest this assay as an inexpensive functional method to predict breast cancer invasive capacity.

## Introduction

Despite critical improvements in treatment and a strong trend towards early diagnosis in developed countries, breast cancer continues to be a leading cause of death worldwide. Almost all such deaths result from breast cancer metastasis to distant organs whose critical functions are compromised. This cancer progression occurs in several stages, but all localized breast cancers that become metastatic must invade locally before the intravasation that leads to metastasis to distant sites. That local invasion occurs first through the thin layer of basement membrane composed primarily of collagen IV and laminins that surrounds tumors and then through the dense extracellular matrix of the breast that is dominated by the presence of fibrillar collagen I. Given that localized breast cancers can only become metastatic if they can breach the basement membrane and invade collagen I-rich environments, either basement membrane or collagen I may be an appropriate *in vitro* environment in which to assess a breast cancer’s ability to invade. Many studies on normal and pathological breast cell development are performed in three-dimensional (3D) environments of basement membrane extract, also known as laminin-rich extracellular matrix (lrECM) [1–13]. These studies follow from pioneering work on breast cancer that was crucial in establishing the importance of cellular microenvironment and specifically, dimensionality on cell behavior [14–17].

Several years ago, a promising assay to identify breast cancer cells with invasive capacity that utilized 3D lrECM was reported [18–20]. This work correlated cell aggregate morphology in 3D lrECM with gene expression signatures [18, 21]. While cells cultured on two-dimensional (2D) plastic were reported to appear nondescript, cell aggregates allowed to develop in 3D lrECM formed one of four morphological classes: stellate, grape-like, mass, or round [18]. This study evaluated 25 available cell lines and showed that aggregate morphology–from most (stellate) to least (round) aggressive–correlated with some measures of cell invasive capacity, primarily the Transwell invasion assay in which cells migrate through a pore-bearing membrane along a nutrient gradient. Moreover, this work showed that cells with similar aggregate morphologies frequently were grouped in hierarchical gene clustering, which itself has been shown to have some prognostic significance [22, 23]. These observations suggested the utility of 3D aggregate morphology as a proxy for cell invasive capacity, possibly with translational value.

We assessed whether aggregate morphology correlated with invasive capacity in *in vitro* assays beyond the Transwell assay. In particular, we investigated correlation between cell aggregate morphology and multicellular invasion in 3D collagen I matrices that recapitulate key biophysical aspects of the *in vivo* stromal breast tissue. In spite of the rich history of using lrECM in breast cancer cell studies and the promising assay described above, collagen I-rich environments may be more appropriate settings in which to study key events in breast cancer progression [24]. Indeed, accumulating evidence shows that density and particular organization of collagen I is causally related to both breast cancer risk and poor prognosis [25, 26]. Moreover, a tumor associated collagen signature (TACS-3) characterized by bundled collagen fibers aligned perpendicular to the tumor/stromal boundary was recently shown to correlate with poor patient outcome [26–32].

We investigated morphological characteristics and dynamic behavior of six cell lines that had been reported to adopt either stellate (MDA-MB-231, Hs 578T, and MDA-MB-157) or grape-like (MDA-MB-468, ZR-75-1, and MDA-MB-453) aggregate morphologies in lrECM in earlier work [18]. We examined whether morphology on 2D or in 3D predicts migratory capacity in two contexts. Specifically, we performed cell morphology assays in isolation and in aggregate on 2D glass and in 3D lrECM or collagen I environments followed by 2D migratory and 3D traction generation and invasion assays. This study reveals that while 2D morphology in aggregate (and in some cases in isolation) is sufficient to predict 3D morphology in both isolation and aggregate, 3D aggregate morphology is not predictive of invasive capacity in 3D collagen. Examples of cells with discordance in stationary and migratory phenotype were identified, with one cell line with stellate aggregate morphology lacking invasive capacity and one cell line demonstrating grape-like aggregate morphology showing high invasive capacity in collagen I environments. We suggest invasion in collagen I environments can predict ability to invade *in vivo*.

## Materials and Methods

### I. Cell Lines and Reagents

MDA-MB-231, Hs 578T, MDA-MB-157, MDA-MB-468, ZR-75-1, and MDA-MB-453 breast cancer cell lines were obtained from the American Type Culture Collection (Manassas, VA). DMEM classical liquid media, RPMI-1640 liquid media, phosphate buffered saline, and fetal media, phosphate buffered saline, and fetal bovine serum (FBS) were obtained from GE Healthcare HyClone (Logan, Utah). Accutase and 100x penicillin-streptomycin-amphotericin B were obtained from MP Biomedicals (Santa Ana, CA). Bovine serum albumin (BSA) was obtained from Thermo Fisher Scientific (Waltham, MA). Nutragen pepsin-treated (PT) bovine collagen type I at 5.9–6.0 mg/mL solution was obtained from Advanced BioMatrix (San Diego, CA). Matrigel growth factor reduced basement membrane matrix (Engelbreth-Holm-Swarm tumor extract–lrECM) at 9.2–10.3 mg/mL solution, 100x MEM nonessential amino acid solution, and Cell Recovery Solution were obtained from Corning (Corning, NY). 10x DMEM solution, 1.0 M HEPES solution, 7.5% sodium bicarbonate solution, and 1.0 N sodium hydroxide solution were purchased from Sigma Aldrich (St. Louis, MO). DMEM without phenol red and AlexaFluor 568 phalloidin was obtained from Life Technologies (Grand Island, NY). 10% buffered formalin phosphate was obtained from Fisher Scientific (Pittsburgh, PA). Triton X-100 detergent was obtained from EMD Millipore (Billerica, MA). The following primary antibodies were employed: α_1_ and α_2_ (Millipore), β_1_ (Beckman Coulter, Brea, CA), α_10_ (Santa Cruz Biotechnology, Dallas, TX), α_11_ (Thermo Fisher Scientific) integrin subunits and α_2_β_1_ integrin (Abcam, Cambridge, MA), E-cadherin (Thermo Fisher), and N-cadherin (Thermo Fisher). FITC-conjugated secondary antibodies were obtained from Thermo Fisher Scientific and Abcam.

### II. Cell Culture Conditions

MDA-MB-231, Hs 578T, MDA-MB-468, and MDA-MB-453 cell lines were maintained on tissue culture plastic in DMEM growth medium supplemented with 10% (v/v) FBS, 1% (v/v) 100x penicillin-streptomycin-amphotericin B, and 1% (v/v) 100x nonessential amino acid solution in a humidified incubator at 37°C with 5% carbon dioxide. MDA-MB-157 and ZR-75-1 cells were similarly maintained in RPMI-1640 growth medium containing the same supplements. Cells were subcultured up to 20 passages.

### III. 2D and 3D Isolated and Aggregate Morphological Assays

Morphological assays were performed with all six cell lines on 2D and in 3D for both isolated cells and cells in aggregate. For isolated and aggregate cells on 2D, cell suspensions of 0.8–4.0 ×10^5^ cells/mL were added to Fluorodishes (World Precision Instruments, Sarasota, FL) and allowed to adhere and grow for 24 hours at 37°C and 5% CO_2_ prior to fixation and imaging as described below.

For isolated and aggregate cells in 3D, lrECM was used in the original concentration of approximately 10 mg/mL and 1 mg/mL collagen gel solution was prepared by diluting stock solution with 10% (v/v) 10x DMEM, 2.5% (v/v) HEPES, 2.5% (v/v) sodium bicarbonate, water and cell suspension in growth medium. All solutions were prepared at 4°C. The pH of collagen I gels was adjusted to 7.4 with sodium hydroxide before the addition of cell suspension. 100 μL of cell-loaded solution was added to a 5 mm glass cylinder affixed to a Fluorodish and the solution was transferred immediately to an incubator at 37°C. For aggregates, 100 μL of cell-free solution was added to a cylinder and 1 μL from a cell pellet was distributed into the solution before transferring the sample to an incubator at 37°C. Nylon mesh was wrapped around the inner circumference of the cylinder prior to adding solution to anchor the gel. Solutions were allowed to gel for 45 minutes, at which point gels were overlaid with growth medium. Additional medium was added to the dish surrounding the glass cylinder to prevent drying during the 24 hour incubation prior to fixation.

### IV. Immunocytochemical Staining and Imaging

Cell morphological assays were performed by fixing and staining cells prior to imaging them with confocal fluorescence microscopy. For phalloidin staining, cells were fixed in formalin phosphate for 10 minutes at room temperature, washed with PBS, permeabilized with 0.5% Triton X for 10 minutes, and washed with PBS again before fluorescently labeled phalloidin was added to stain F-actin in the cells. The samples were incubated overnight at 4°C and washed with PBS before imaging. Imaging was done on an Olympus Fluoview 300 confocal laser scanning microscope using a 543 nm excitation laser, 570 nm long pass mirror, and 60x oil objective. For cadherin staining, cells were fixed in formalin phosphate for 10 minutes at room temperature, washed with PBS, blocked for nonspecific staining with BSA for 30 minutes, and washed with PBS again before primary antibody for E- or N-cadherin was added to the cells for 1 hour at room temperature. Cells were then washed with PBS and FITC-conjugated secondary antibody was added. The cells were left overnight at 4°C and washed with PBS before imaging. Imaging for E- and N-cadherin was done using a 488 nm excitation laser, 570 nm short pass mirror, 510 nm bandpass filter, and 60x oil objective. For all samples, maximum intensity projections for each cell were created using ImageJ from images taken every 2 μm in the z-direction.

### V. Fluorescence-activated cell sorting

Cells were detached from the cell culture plate with Accutase, resuspended in phenol red free DMEM with HEPES and 10% FBS, divided into tubes for staining, and kept on ice for the remainder of the procedure. Cells were washed with PBS containing 1% FBS in between steps, left on ice for 1 hour in primary antibody and then for 40 minutes in secondary antibody. Finally, cells were resuspended in PBS and measurements were taken on a flow cytometer (Becton Dickinson FACSCalibur with BD CellQuest Pro software).

### VI. 2D Migratory Assay

Ibidi culture-inserts (Verona, WI) were used for the 2D migratory gap assay. All cell lines were plated at 1×10^5^ cells in 80 μL in each well and incubated at 37°C and 5% CO_2_ except for Hs 578T cells, which were plated at 1×10^4^ cells per well. This was necessary because Hs 578T cells were larger and flatter than the other cell lines when grown on 2D and fewer cells were required to form a monolayer on a given area. When the cells reached confluence, medium in the wells was changed to DMEM and 2% (v/v) FBS. The culture-insert was removed 24 hours later and the cells were washed with regular growth medium until the gap between the two confluent areas was clear of any cells. Laser scanning transmittance images were taken immediately after washing and at 6, 24, and 48 hours on the Olympus Fluoview 300 using 488 nm excitation and a 10x air objective. Data were averaged from two independent experiments performed in biological triplicate.

### VII. 3D Gel Contraction Assay

500 μL of 1 mg/mL collagen I gels containing 5×10^5^ cells/mL were prepared as for the 3D isolated cell morphological assays and plated on the 23 mm coverslip bottom of 35 mm Fluorodishes. Gels were incubated at 37°C for 60 minutes, overlaid with 2 mL cell culture medium, and manually released from the coverslip bottom. Gels were allowed to contract for 4 hours. Images were taken upon release and 4 hours after release using digital photography. Contraction is expressed as a percentage decrease of gel area. Data were averaged from two independent experiments performed in biological triplicate.

### VIII. 3D Spheroid Invasion Assay

Spheroids were formed using low adhesion lipidure-coat plates from NOF America Corporation (White Plains, NY). 200 μL of 1×10^4^ cells/mL cell suspension and 0.25 mg/mL lrECM in growth medium were plated in the wells of the plate. The plate was centrifuged at 4°C for 10 minutes and then incubated at 37°C and 5% CO_2_ for 24 hours. Spheroids were transferred to Cell Recovery Solution for 45–60 minutes before implantation in collagen.

To prepare collagen gels with a single spheroid, 1 mg/mL collagen gel solutions were prepared by diluting collagen stock solution with 10% (v/v) 10x DMEM, 2.5% (v/v) HEPES, 2.5% (v/v) sodium bicarbonate, and water. To prevent self-assembly of collagen monomers, all solutions were prepared at 4°C. Sodium hydroxide was added to adjust the pH to 7.4. 200 μL of neutralized collagen solution was added to a 5 mm glass cylinder glued to a Fluorodish and a spheroid was added in 5 μL growth medium. The sample was transferred immediately to an incubator at 37°C. Nylon mesh around the inner circumference of the cylinder helped to anchor the collagen gel. Collagen solutions were allowed to gel for 1 hour, at which point gels were overlaid with growth medium and the cylinders were surrounded by additional liquid to prevent drying during extended incubation periods. Images were taken 2 hours and 24 hours after implantation. A 488 nm excitation laser and 10x air objective were employed to take scanning transmittance images of spheroid invasion, and the same laser with a 60x oil objective was used to acquire confocal reflectance images of collagen fiber arrangement around the spheroids.

## Results and Discussion

### I. 2D and 3D Isolated and Aggregate Morphology are Correlated

Six breast cancer cell lines were investigated in this work, three that were previously identified as demonstrating stellate aggregate morphology (MDA-MB-231, Hs 578T, MDA-MB-157) and three that were identified as demonstrating grape-like aggregate morphology (MDA-MB-468, ZR-75-1, and MDA-MB-453 cells) in lrECM [18]. Key characteristics of the cell lines employed in this study are shown in Table 1.

**Table 1:** Cell line characteristics.

Cell line	Receptor status* ER PR HER2	Aggregate morphology^+^	Aggregate morphology^#^
MDA-MB-231	- - -	stellate	stellate
Hs 578T	- - -	stellate	stellate
MDA-MB-157	- - -	stellate	stellate
MDA-MB-468	- - -	grape-like	grape-like
ZR-75-1	+ - - /+ + -	grape-like	mass
MDA-MB-453	- - - /- - +	grape-like	grape-like

Characteristics of cell lines investigated: receptor status and aggregate morphology.

*Tumor receptor status: + = positive and— = negative for ER = estrogen receptor, PR = progesterone receptor, and HER2 = human epidermal growth factor receptor 2. In cases where discrepancy between reports is present across publications, both reported receptor statuses are shown [18, 33].

^+^Aggregate morphology in a “3D on top” lrECM assay (cells on an lrECM gel and covered with an additional thin layer of lrECM) as reported by Kenny et al. [18].

^#^Aggregate morphology on 2D glass and in 3D 1.0 mg/mL collagen I matrix from this study.

**Fig 1:**
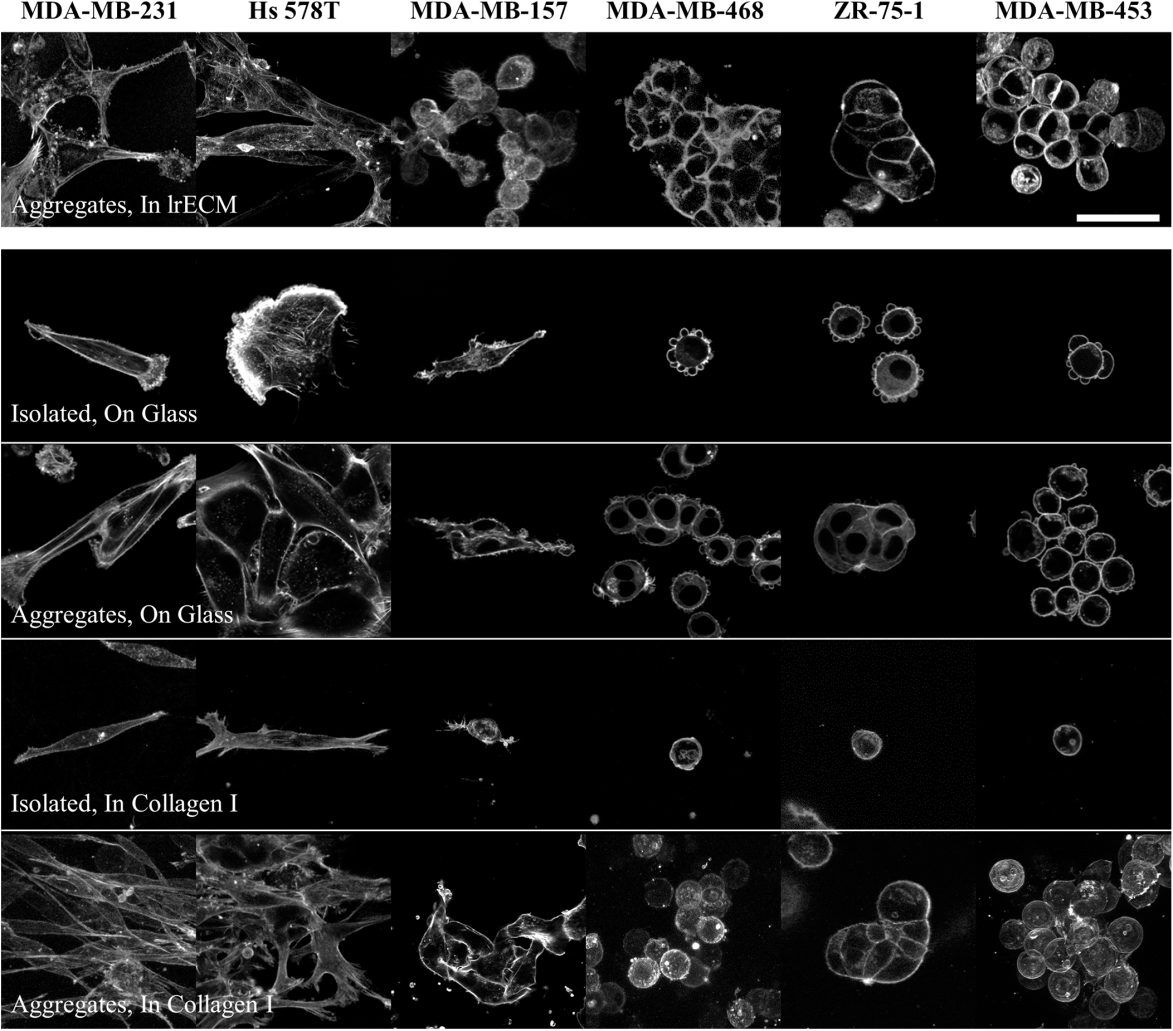
Morphology of representative isolated cells and aggregates of each investigated cell line in 3D 10 mg/mL lrECM, on 2D glass, and in 3D 1.0 mg/mL collagen I matrices. Cells were fixed after 24 hours of culture and stained for F-actin. Scale bar is 50 μm.

Cell aggregate morphology was first analyzed in 3D lrECM. Most cell lines were found to exhibit the same aggregate morphology as reported previously despite a somewhat different approach to aggregate preparation compared to that presented in previously published studies [18, 19] (Fig 1). MDA-MB-231 and Hs 578T cells were stellate in lrECM. MDA-MB-157 cells exhibited some aspects of stellate aggregate morphology, their reported morphology, though their extension was less pronounced than the other two cell lines in this category. Among the cell lines previously reported to form grape-like cell aggregates, MDA-MB-453 cells displayed clear grape-like aggregate morphology while MDA-MB-468 and ZR-75-1 cells displayed aspects of grape-like and mass aggregate morphology, with some flattening of the cells at points of cell-cell contact.

Cells from each cell line were next cultured on 2D glass. MDA-MB-231 and MDA-MB-157 cells cultured at low density on this surface typically displayed an extended spindle-like cell body with protrusions at the leading and trailing edges, while Hs 578T cells were flatter, less anisotropic, and showing lamellipodial ruffling. Cells of these three cell lines retained similar morphologies when cultured at higher densities that allow for cell-cell contact and aggregate formation, with the MDA-MB-231 and MDA-MB-157 cells showing significant alignment along their long axes, a signature of stellate aggregate morphology. Cells previously identified as grape-like in 3D lrECM culture were round and demonstrated bleb-like protrusions when cultured in isolation on the 2D substrates. At higher density on these substrates, MDA-MB-453 and MDA-MB-468 cells displayed classical grape-like aggregate morphology while ZR-75-1 cells demonstrated mass aggregate morphology.

In 3D 1.0 mg/mL collagen gels, MDA-MB-231 cells displayed very similar appearance both in isolation and in aggregate to that displayed on the 2D substrate. Hs 578T cells in 3D collagen adopted a thinner, more extended morphology in isolation in 3D collagen I than on 2D. In aggregate these cells demonstrated stellate aggregate morphology in 3D collagen I although there was somewhat less alignment along the long axes of the cells than was obvious in the MDA-MB-231 aggregates. Like MDA-MB-231 cells, MDA-MB-157 cells looked very similar when cultured on 2D and in 3D collagen I. While these cells were anisotropic, there was less extension than in the other two stellate cell lines described and less alignment along a particular direction. The three cell lines that demonstrated round morphology in isolation when cultured on 2D continued to do so when cultured in 3D collagen I. They also demonstrated very similar aggregate morphology on 2D and in 3D culture, with the MDA-MB-453 and MDA-MB-468 cells demonstrating typical grape-like aggregate morphology, and ZR-75-1 demonstrating mass aggregate morphology.

In sum, the three cell lines investigated that had been identified as demonstrating stellate aggregates in 3D lrECM also do so in 3D collagen I. Two of the three cell lines that had previously been identified as demonstrating grape-like aggregate morphology in lrECM do so in 3D collagen I, while one (ZR-75-1) yields mass aggregates. In all cases, aggregate morphology displayed by cells cultured in 2D on glass is a good predictor of 3D aggregate morphology in collagen I matrices. As such, if 3D aggregate morphology is a predictor for invasive capacity, the even simpler assay of aggregate morphology on 2D may be similarly predictive.

### II. Isolated and Aggregate Morphologies are Uncorrelated with 2D Migratory Behavior

The correlation between isolated and aggregate cell morphology and migratory speed was tested in 2D gap assays. Migratory mode (collective vs. individual migration) and speed of gap closure may be expected to correlate with aggregate morphology as both depend in part on the number and strength of cell-cell and cell-environmental contacts.

**Fig 2:**
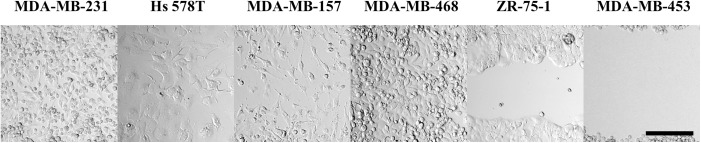
Transmittance images of cells from each cell line investigated in a gap assay at 24 hours. Initial gap distance is 500 μm, the height of the images. Scale bar is 200 μm.

Among cells with stellate aggregate morphology in 3D collagen I and on 2D tissue culture treated glass, the MDA-MB-231 cells migrated most rapidly. After 6 hours, the cell front of the MDA-MB-231 monolayers had closed substantially, with select cells having reached the center of the gap, and by 24 hours, the gap was completely filled in (Fig 2). The Hs 578T and MDA-MB-157 cells also migrated individually, though more slowly than the MDA-MB-231 cells. Some cells had spanned the gap by 24 hours but the gap was not completely filled in by that time point.

Cell lines that adopted round morphology in isolation and demonstrated grape-like or mass morphologies in aggregate showed more discrepancy in migratory speed and migratory mode. MDA-MB-468 cells closed the gap very quickly, at a rate similar to MDA-MB-231 cells. Individual cells spanned the gap by 6 hours and the gap was fully filled in by 24 hours. ZR-75-1 cells migrated collectively, in a sheet-like front. These cells required more than 48 hours to close the gap. MDA-MB-453 cells did not appear to migrate, but did proliferate minimally into the gap over 48 hours.

In sum, while we found strong correlation between the 2D and 3D morphologies, both in isolation and aggregate, we found limited correlation between aggregate morphology and migratory speed in the gap assay. The MDA-MB-231 and MDA-MB-468 cells were the fastest to traverse the gap though they are from different aggregate morphology classes. Moreover, cell lines that form grape-like aggregates represented cells both among the fastest (MDA-MB-468) and slowest (MDA-MB-453) in this assay.

### III. Isolated and Aggregate Morphologies are Uncorrelated with 3D Invasion in Collagen

While no correlation between morphology and 2D migratory speed was observed, given that correlation was seen previously between aggregate morphology and invasive capacity in Transwell assays, it is possible that the dimensionality of the migratory or invasive assay is critical in revealing correlations between cell morphology and invasive behavior. We assessed 3D invasive capacity through spheroid invasion assays in collagen I environments. In such assays, both cell-cell and cell-environmental contacts found *in vivo* are recapitulated.

The ability of cell lines to form spheroids speaks in part to their capacity to form tight cell-cell adhesions. Interestingly, cells that demonstrate stellate aggregate morphology tend to form tight spheroids well. In the most commonly used method to produce spheroids, hanging drop culture, while all three stellate cell lines formed compact spheroids, none of the other cell lines did. As such, a different approach to generate spheroids was used that was suitable for all cell lines, as described in Materials and Methods [34].

**Fig 3:**
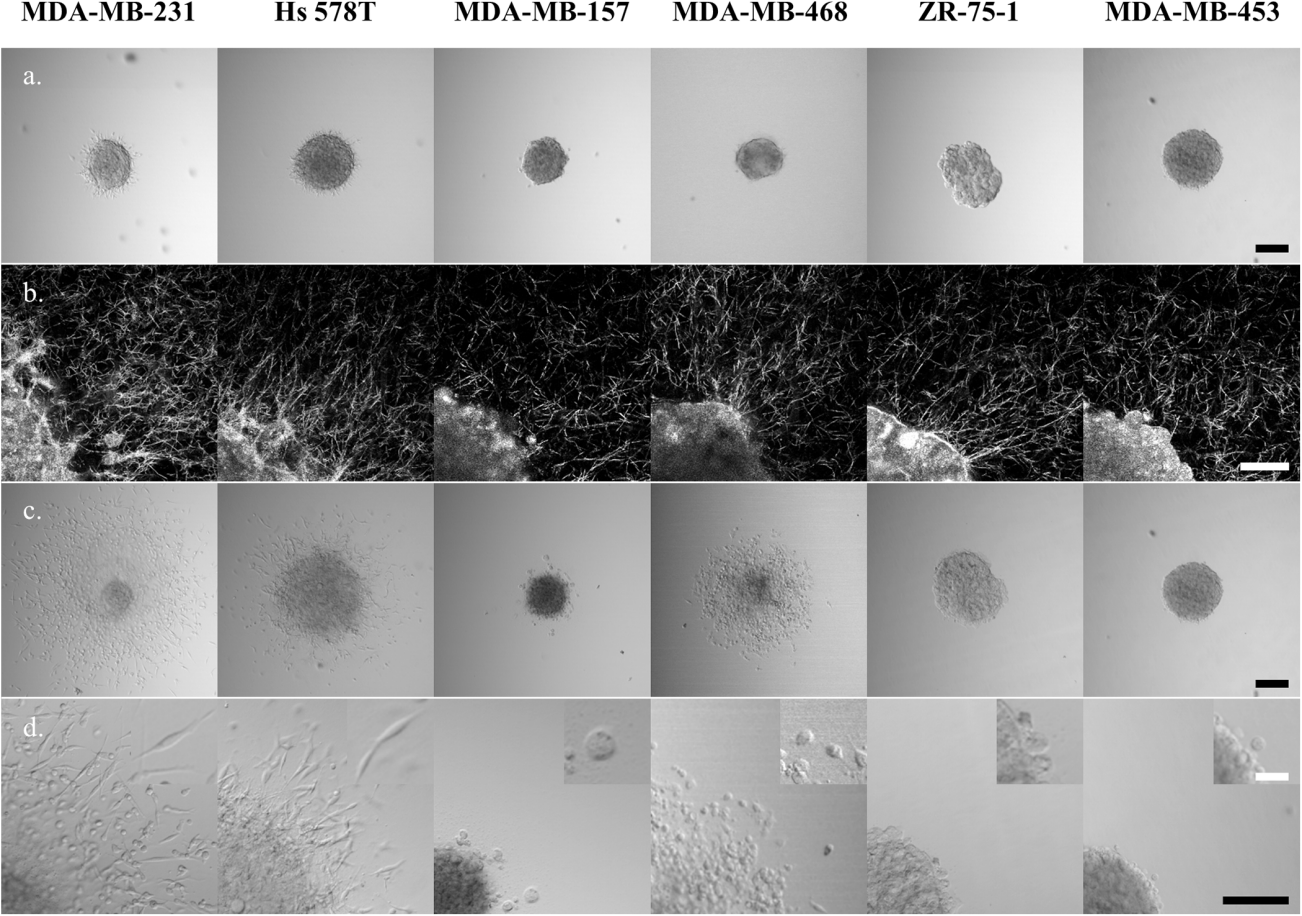
Spheroids of each investigated cell line at 2 hours and 24 hours after implantation in 1.0 mg/mL collagen I. (a) Transmittance images at 2 hours, (b) confocal reflectance images of collagen surrounding the spheroids (and a portion of the spheroid in the lower left corner) at 2 hours, (c) transmittance images at 24 hours, (d) magnified view of cells invading from the spheroid at 24 hours. Additional magnification shown in the inset highlights invading cell morphology. Black scale bars are 200 μm and white scale bars are 50 μm. Confocal reflectance images (row b) have had an optical artifact that causes a bright spot in the center of the field of view removed by replacement of this spot with a representative area from elsewhere in the image.

Images of representative spheroids at 2 and 24 hours after implantation into the collagen I gels are shown in Fig 3. At the two hour time point, initial invading cells were seen in MDA-MB-231 and Hs 578T spheroids (Fig 3A). At this same time point, evidence of cells generating traction on surrounding collagen was seen in most cell lines, as evidenced by collagen fibers aligned radially near the spheroid edge. This alignment was seen not only in the cell lines with early onset of invasion (at two hours) but also in MDA-MB-157, MDA-MB-468, and ZR-75-1 spheroids. Only the MDA-MB-453 spheroids appeared surrounded by an isotropic matrix at this time point.

Three of the cell lines investigated were found to substantially invade 3D collagen I matrices within 24 hours after spheroid implantation, MDA-MB-231, Hs 578T, and MDA-MB-468, the first two of which form stellate and the last grape-like aggregates. Of all the cell lines investigated, when prepared as spheroids the MDA-MB-231 cells were most invasive, both in terms of cell speed and invasive cell density. Moreover, the original spheroid core largely disassembled as cells from that core disseminated into the surroundings, indicative of particularly aggressive invasive behavior. The invasive MDA-MB-231 cells primarily adopted a stellate morphology during invasion, and many cells invaded a distance of 400 μm within 24 hours, a rate of approximately 0.3 μm/min, similar to that reported previously for single cells in a similarly dense collagen I environment [35].

The other cell lines with stellate aggregate morphology displayed different invasive behaviors. Hs 578T spheroids demonstrated substantial invasion with cells exhibiting primarily stellate morphology, invading moderately quickly and at moderate density in comparison to the MDA-MB-231 cells. Unlike in MDA-MB-231 spheroids, the dense spheroid core did not disseminate but instead expanded substantially over the 24 hours. The third cell line with stellate aggregate morphology, MDA-MB-157, demonstrated very limited invasion with only a few (round) cells dispersing from the spheroid core.

The cell lines that adopted round morphology in isolation also displayed a diversity of invasive behaviors. MDA-MB-468 spheroids invaded efficiently, with round but polarized cells that dispersed approximately as quickly as Hs 578T cells and as densely as MDA-MB-231 cells into the surrounding collagen matrix. In contrast, ZR-75-1 cells and MDA-MB-453 cells showed no clear evidence of invasion in these collagen I environments.

### IV. Cell Contractility Is Necessary but Insufficient to Predict Invasion in Collagen Surroundings

In the spheroid assay, of the cells with stellate aggregate morphology, MDA-MB-231 and Hs 578T cells invaded successfully. The invasive cells of both cell lines demonstrated typical mesenchymal invasion, with strongly polarized morphology and a motility cycle in which tractional forces were generated on their surroundings before the cell body was pulled forward. The tractional force generation was evidenced by remodeling of surrounding collagen fibers into radial patterns around the spheroid within several hours of spheroid implantation and before notable invasion (Fig 3B). Of the three cell lines in which cells demonstrated round morphology in isolation and grape-like or mass morphology in aggregate, again there was no clear correlation between aggregate morphology and invasion in spheroid assay. Among these cells, MDA-MB-468 cells invaded the collagen gels with similar speed to the Hs 578T cells and similar density to the MDA-MB-231 cells. The other three cell lines showed limited or no invasion in the spheroid assay. The MDA-MB-468 cells migrated with a different phenotype than the MDA-MB-231 and Hs 578T cells, exhibiting round morphology during the majority of the migratory cycle and bleb-based protrusions instead of the filopodia and related actin-driven protrusions seen in typical mesenchymal invasion. Despite this bleb-mediated migratory mode, the cells did appear to pull on collagen fibers in their environment (Fig 3B), suggesting that traction generation is also important in this migratory mode. These observations suggested that ability to generate traction on and reorganize collagen fibers is a necessary activity to allow for invasion in the spheroid assay. To test this hypothesis, integrin profiling and collagen contractility assays were performed.

**Fig 4:**
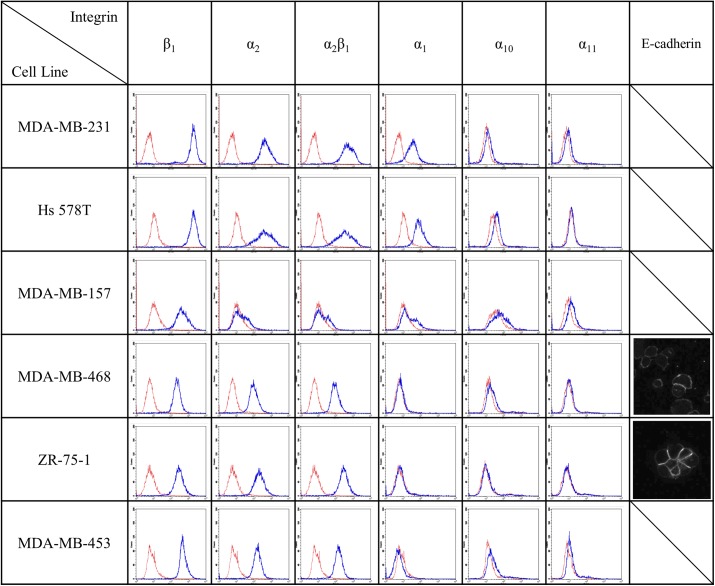
FACS based analysis of integrin receptors relevant to collagen binding. Blue line shows integrin fluorescence intensity and red line shows secondary antibody control fluorescence intensity. Immunocytochemical staining for E-cadherin is shown in the rightmost column for the two cell lines that showed detectable levels in this assay.

The integrins that preferentially bind to collagen contain the β_1_ subunit. As measured via fluorescence-activated cell sorting (FACS), all cell lines assessed in this study have significant amounts of β_1_ integrin, with particularly high levels seen in the MDA-MB-231 and Hs 578T cells (Fig 4). Most cell lines also have significant amounts of the α_2_ subunit that pairs with the β_1_ subunit to form the α_2_β_1_ integrin that is the functional receptor for collagen I fibrils [36]. The exception is the MDA-MB-157 cell line. In all cases, the level of α_2_ subunit was limiting and paralleled the detected level of α_2_β_1_ integrins. The presence of significant levels of α_2_β_1_ integrins in all cell lines but MDA-MB-157 suggests that each of the other cell lines has the capacity to generate traction on and reorganize collagen I. We also measured the levels of other subunits that when coupled with the β_1_ subunit may bind to collagen: α_1_, α_10_, and α_11_ [37]. No cell line exhibited high levels of these integrin subunits, though MDA-MB-231 and Hs 578T cells exhibited detectable levels of α_1_ integrins. The integrins that are relevant for collagen I binding are the same ones relevant for binding collagen IV and laminin, the key components of basement membrane and lrECM [38]. As such, integrin requirements for invasion in collagen I environments are expected to be similar to those in lrECM. The results presented in Fig 4 suggest that each cell line, save MDA-MB-157, has the capacity to adhere to and generate traction on collagen I fibrils. Thus, the expression level of suitable integrins did not distinguish invasive from noninvasive cells in the collagen I environment.

**Fig 5:**
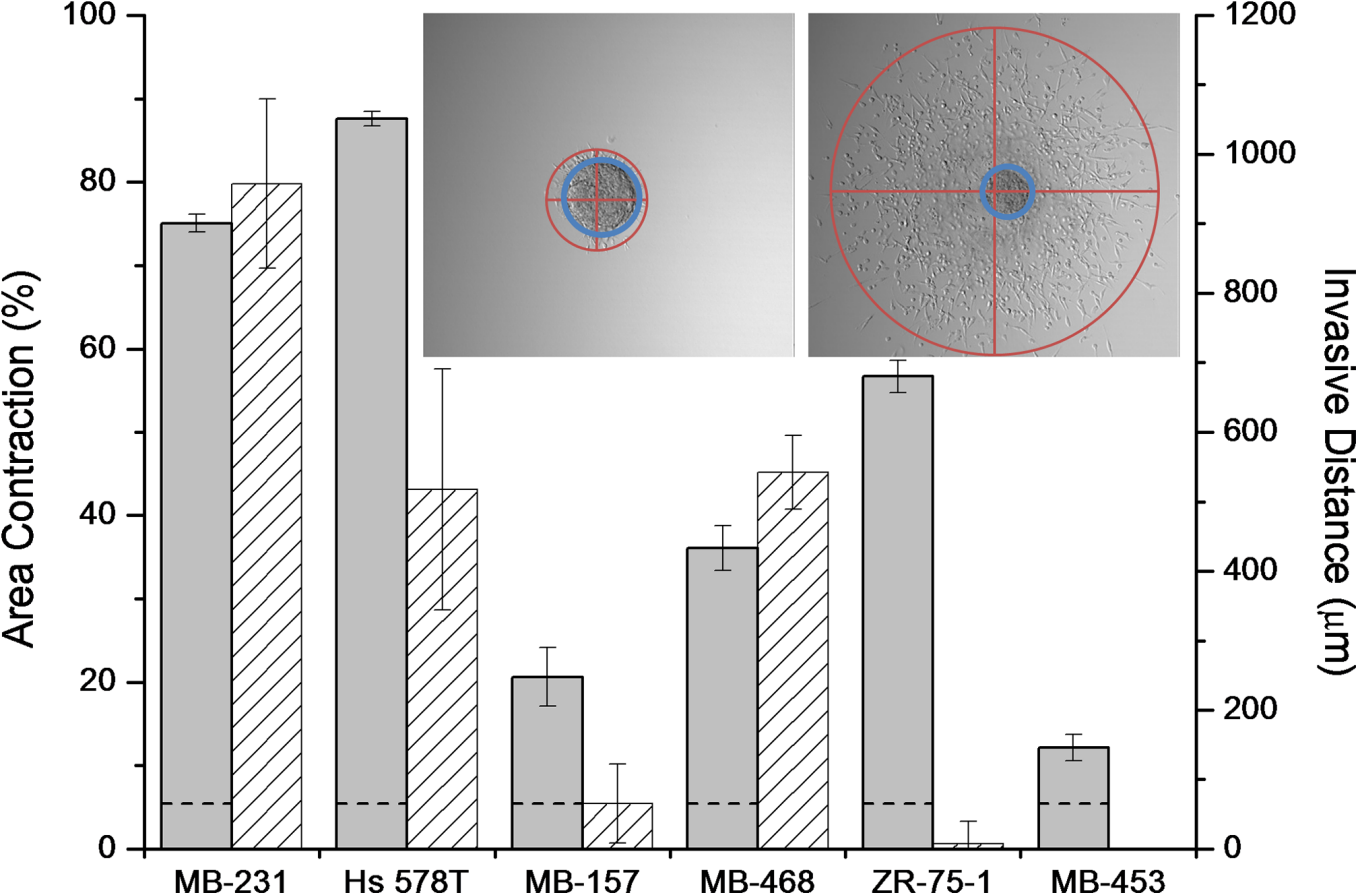
Percent area contraction of collagen I gels with dispersed cells (gray) and invasive distance from spheroids (pattern) for each cell line. Data are average values with error bars indicating standard deviation from three to seven independent experiments each in biological triplicate (for contraction) and 11–18 spheroids in biological replicate (for invasion). Dotted line indicates contraction of collagen I gels without cells. Inset shows extent of invasion (red) and spheroid core (blue) for a representative MDA-MB-231 spheroid. Invasive distance is defined as the diameter of the blue circle (spheroid core) subtracted from the diameter of the red circle (invasion area) drawn at 24 hours. The spheroid core used in the invasive distance calculation is the larger of the spheroid core at either 2 or 24 hours. Only the Hs 578T cell line exhibited a larger core at 24 hours than at 2 hours.

While all cell lines investigated (except MDA-MB-157) have suitable integrins for collagen adhesion and traction generation, it is possible that integrin level does not correlate with integrin function. As such, we also measured the ability of cells from each cell line to generate traction on collagen fibrils through contraction assays. In these assays, dispersed cells were allowed to contract a 1.0 mg/mL collagen gel over four hours. Here, the two stellate cell lines with abundant α_2_β_1_ integrins, MDA-MB-231 and Hs 578T, contracted the gels to less than 30% of their initial size within four hours (Fig 5). Of the cells demonstrating grape-like or mass aggregate morphology, MDA-MB-468 and ZR-75-1 cells were moderately efficient at contraction, resulting in gels approximately half their initial size after four hours, while MDA-MB-453 cells were poor contractors. Interestingly, MDA-MB-157 cells showed some contractile ability both in the contraction assay (Fig 5) and in the alignment of collagen around the spheroid (Fig 3B) despite their low levels of relevant integrins. Overall, the contraction assays show some correlation with aggregate morphology, as the two stellate lines with relevant integrins contract the gel very efficiently while other cell lines with comparable integrin levels either contract moderately or very inefficiently (MDA-MB-453).

We also found some correlation between collagen contraction and collagen invasion as measured in the spheroid assay (Fig 5). The three invasive cell lines were at least moderately contractile in the contraction assay. However, the ZR-75-1 cells were relatively efficient contractors but did not invade in the spheroid assay at all.

Taken together, the results of the spheroid invasion and collagen contraction assays suggest that collagen contractile ability is necessary but insufficient to allow for cell invasion in a collagen I environment. With neither integrin profiles nor contractile ability clearly distinguishing invasive from noninvasive cells, we investigated levels of cadherins in these cell lines. The existence of strong cell-cell contacts could, for example, explain why tractional force generation on collagen fibrils is necessary but insufficient for invasion from spheroid culture into a surrounding collagen I matrix. Altered balance of cell-cell and cell-environmental affinity is a key aspect of the epithelial to mesenchymal transition that plays a critical role in tumor progression and has been invoked previously to explain varied invasive capacity both experimentally and in simulations [39–41]. Previous studies showed high levels of E-cadherin in ZR-75-1 cells, moderate levels in MDA-MB-468 cells, and no detectable E-cadherin in MDA-MB-231, Hs 578T, or MDA-MB-453 cells [18]. Via immunocytochemistry, we confirmed these findings and further found no notable staining in the MDA-MB-157 cells (Fig 4). The high level of E-cadherin expressed by the ZR-75-1 cells may explain why these cells, with appropriate integrins and strong contractile ability, fail to invade in spheroid assay. N-cadherin, which has been shown to contribute to invasive capacity in breast cancer cells, was detectable by immunocytochemical staining only in the Hs 578T cells (data not shown), consistent with a previous report [42].

### V. Cell Invasive Capacity in Collagen I Correlates with Gene Expression Signatures

Given that full correlation was not found between any of the static or dynamic cell signatures considered above and spheroid invasive capacity in collagen I environments, we considered correlation between collagen I invasive capacity and gene expression signatures to potentially capture the wide range of factors involved in invasion both in the *in vitro* collagen I environment and in the *in vivo* extracellular matrix surrounding tumors.

In recent years, gene expression profiling has been used to classify breast tumors in a manner that has prognostic significance. Early molecular categorization via unsupervised hierarchical gene clustering of human breast tumors showed clustering into four categories–luminal, basal, ERBB2 (the gene associated with HER2 expression), and normal [22], while later studies stratified luminal tumors and identified another category known as claudin-low [43, 44]. Smaller gene sets have since been used to predict recurrence and tailor treatment for women with early stage breast cancers, with a 70 gene set appropriate for both hormone receptor positive and negative cancers and a 21 gene set appropriate for predicting recurrence in hormone receptor positive tumors [23, 45]. Both classifiers include genes involved in proliferation and the epithelial to mesenchymal transition as well as genes that are outside categories obviously associated with cancer development and progression.

More recently, gene profiling and unsupervised hierarchical clustering have been performed on commonly used breast cancer cell lines to understand their similarities and differences from primary breast tumors and to identify cell lines that may be useful in addressing particular questions [21, 46]. Two studies each examined gene expression in more than 50 breast cancer cell lines. Hierarchical clustering of gene expression profiles of these cell lines revealed somewhat different groupings than did profiling of primary breast tumors. Like tumors, cell lines clustered broadly into luminal and basal categories, though the particular categories established differed somewhat from the tumor categories and were termed luminal, basal A and basal B [21, 46]. These two studies identified each of the stellate cell lines investigated here as members of the basal B class, while MDA-MB-468 was categorized as basal A and the ZR-75-1 and MDA-MB-453 cell lines were categorized as luminal. Kao et al. and Prat et al. also used breast tumor classifications to classify the cell lines, with all stellate cell lines clustered in either of the aggressive tumor classifications of claudin-low or ERBB2, the latter despite the fact that none of these cell lines are HER2 positive [46, 47]. The grape-like cell lines were not found to all belong to the same category, with both studies identifying MDA-MB-468 cells as basal and MDA-MB-453 cells as either luminal B or luminal. ZR-75-1 cells were categorized as luminal A and luminal, by Kao et al. and Prat et al., respectively. Kao et al. also assessed the breast cancer cell lines in terms of whether they displayed a positive wound healing signature (512 genes), a positive hypoxia signature (123 genes), or poor prognosis as assessed by the 70 gene assay described above [23, 46, 48, 49]. We note that each of these signatures was performed for cells cultured on 2D and that cells cultured in 3D, and particularly in 3D collagen I, may yield somewhat different results [18]. Five of the six cell lines we studied have also been assessed for percentage of cancer stem cells (CSCs), which may reasonably be expected to correlate with invasive and metastatic capacity [50]. Each of these cell lines was found to have measurable but low levels of CSCs as assessed through aldehyde dehydrogenase expression and activity, with less than 1% found in the most invasive MDA-MB-231 cell line. However, not all CSC markers correlate and, like other gene expression profiles, these markers may depend on the dimensionality of cell culture [51]. Table 2 summarizes results for the cell lines investigated in this study.

**Table 2:** Cell line morphological, genetic, and functional characteristics.

Cell line	Aggregate morphology^#^	Cancer stem cell (%)^§^	Cell line subtype*	Tumor subtype**	Tumor subtype***	Gene signatures^+^ for Wound · Hypoxia · 70 Gene	Collagen contractile capacity^#^	Collagen relevant integrins^#^	Collagen invasion capacity^#^
MDA-MB-231	stellate	0–1%	BB	ERBB2	CLL	+ + +	+	+	+
Hs 578T	stellate	0–1%	BB	ERBB2	CLL	+ + -	+	+	+
MDA-MB-157	stellate	1–5%	BB	ERBB2	CLL	-—-			
MDA-MB-468	grape-like	N/A	BA	B	B	-—+	+	+	+
ZR-75-1	mass	1–5%	L	LA	L	-—-	+	+	
MDA-MB-453	grape-like	1–5%	L	LB	L	-—-		+	

Cell line aggregate morphological classes, cancer stem cell (CSC) percentages, subtypes, positivity for wound, hypoxia, and 70 gene signature, and collagen related activities.

^§^CSC percentages obtained from Ref. [50]. MDA-MB-468 cells were not included in this study and thus labeled N/A (not available).

*Subtypes of breast cancer cell lines identified by unsupervised hierarchical clustering: BB = basal B, BA = basal A, L = luminal [21, 46].

**Subtypes of breast cancer cell lines using tumor cell classifications described in Ref. [43] as reported in Ref. [46]: ERBB2 = ERBB2 signature; B = basal, LA = luminal A, LB = luminal B. ***Subtypes of breast cancer cell lines using tumor cell classifications as reported in Ref. [47]: CLL = claudin-low; B = basal; L = luminal.

^+^+ = positive and— = negative for wound or hypoxia gene signatures or poor prognosis signature in the 70 gene assay as reported in Ref. [46].

^#^This study: + indicates moderate to high collagen contractile capacity (Fig 5), moderate to high levels of collagen-relevant integrins (Fig 4), and capacity to efficiently invade collagen I gels from spheroid culture (Figs 3 and 5).

Among the cell lines tested here, stellate cell aggregate morphology clustered strongly with gene expression profiles as determined either from cell lines or tumors. Such correlations were not as strong in the other cell lines investigated. Among the functional wound-healing and hypoxic gene signatures that have been previously associated with cancer prognosis, two of the three stellate cell lines demonstrated a positive response while none of the grape-like cell lines did. Given the importance of collagen I integrins in wound healing, it is perhaps unsurprising that the two cell lines found to be very efficient contractors of collagen I show positive wound healing signature. Intriguingly, the poor prognosis prediction from the 70 gene assay does not correlate with aggregate morphology nor with the categorizations assigned by unsupervised clustering. However, it does predict poor prognosis in two of the three cell lines found to be invasive in this study, including the MDA-MB-468 cell line that would not be predicted to be aggressive from aggregate morphology, integrin profile, or contractile capacity. This finding suggests the collagen invasion assay, or a related one based on primary tumors or organoids, as an inexpensive functional assay to identify tumors with collagen invasive capacity and poor prognosis.

## Conclusion

We found that aggregate morphology of six breast cancer cell lines within 3D collagen I gels was very similar to that in lrECM, though we categorized ZR-75-1 as exhibiting mass aggregates rather than grape-like aggregates. Unlike in previous studies, we found that in some circumstances cell aggregate morphology in 3D physiologically relevant environments could be predicted from cell morphology in simple contexts. Indeed, aggregate cell morphology on 2D in all cases investigated was very similar to that seen in 3D collagen I and 3D lrECM. Additionally, cell morphology in isolation either in 3D collagen I or on 2D glass was sufficient to establish which cells would adopt a stellate aggregate morphology. Despite the ability to predict 3D aggregate morphology from morphology in simpler contexts, aggregate morphology was not found to fully correlate with cell migratory behavior, collagen contractile behavior, balance of integrin and cadherin expression, or invasion from a spheroid into a collagen I gel. Three of the six cell lines investigated here–MDA-MB-231, Hs 578T, and MDA-MB-468 –were found to be invasive in collagen I gels. Two of these cell lines were from the stellate aggregate morphology class and one was from the grape-like aggregate morphology class. The invasive cell lines were not found to be distinguishable from noninvasive cell lines through consideration of aggregate morphology, integrin and cadherin expression, or collagen contractility capacity, though collagen contractility appears to be necessary but insufficient to predict collagen I invasive capacity. For the set of cell lines studied, all cell lines that demonstrated stellate aggregate morphology and had suitable integrins for collagen I attachment were invasive. For cell lines that demonstrated other aggregate morphologies, no clear predictor was found. However, it is intriguing that two of the three invasive cell lines, including the MDA-MB-468 cells from the grape-like morphological aggregate class, were associated with poor prognosis signature in a validated 70 gene assay. This suggests that the spheroid invasion assay in collagen I recapitulates key aspects of the *in vivo* tumor environment beyond cell-cell and cell-environmental contacts. This finding also implies that spheroid invasion or related assays can serve as functional assays to identify breast cancers with invasive capacity and poor prognosis, including those that do not have morphological markers of aggressiveness.
